# Draft Genome Sequence of a Novel Marine Bacterium, *Paraglaciecola* sp. Strain S66, with Hydrolytic Activity against Seaweed Polysaccharides

**DOI:** 10.1128/genomeA.00304-16

**Published:** 2016-04-21

**Authors:** Mikkel Schultz-Johansen, Mikkel A. Glaring, Pernille K. Bech, Peter Stougaard

**Affiliations:** Department of Plant and Environmental Sciences, University of Copenhagen, Frederiksberg, Denmark

## Abstract

A novel agarolytic gammaproteobacterium, *Paraglaciecola* sp. S66, was isolated from marine samples of eelgrass (*Zostera* sp.) and sequenced. The draft genome contains a large number of enzyme-encoding genes with predicted function against several complex polysaccharides found in the cell walls of algae.

## GENOME ANNOUNCEMENT

Marine polysaccharides present in algae and marine animals require the action of specialized carbohydrate-active enzymes for their disassembly (CAZymes, http://www.cazy.org). Although many CAZymes targeting polysaccharides of terrestrial origin have been described, knowledge of the catalytic machineries degrading the complex marine polysaccharides is still limited. Agars and carrageenans are structural cell-wall polysaccharides of red seaweeds and are routinely used in solid growth media in laboratories and as stabilizers in conventional food and cosmetic products. Enzymatic degradation of these polysaccharides has recently received attention due to the potential bioactive properties of agaro- and carrageenan-oligosaccharides (1, 2). Agarolytic bacteria are common within the family *Alteromonadaceae* of the *Gammaproteobacteria*. The genus *Paraglaciecola*, recently reclassified from *Glaciecola* (3), currently consists of eight described type strains, at least two of which produce agarases: *P. agarolytica* (4) and *P. mesophila* (5). In this report, we describe the draft genome sequence of a new agarolytic species of *Paraglaciecola*. Insight into the genomes of bacteria capable of degrading marine polysaccharides may aid our understanding of carbon cycling in marine environments and serve as a source of novel carbohydrate-degrading and -modifying enzymes for industrial applications.

*Paraglaciecola* sp. S66 was isolated from marine samples of eelgrass (*Zostera* sp.) collected at the coast of Northwest Zealand, Denmark, and was identified based on the ability to visibly degrade the agar growth medium. Genomic DNA was extracted from a liquid culture grown at 20°C in marine broth and sequenced on an Illumina HiSeq platform producing 150-bp paired-end reads from a short insert library. Assembly was performed using Velvet and Velvet optimizer (6). The result was 128 contigs organized in 20 scaffolds with an *N*_50_ value of 529,023 bp. The size of the draft genome is 5,221,450 bp with a G+C content of 42.15%. The genome was annotated using GLIMMER-3 on the RAST server (7), resulting in 4,703 predicted coding sequences and 54 RNA genes.

A large number of CAZymes were identified in the genome of S66 based on an HMMsearch (*E* value cutoff 1.0e-5) against a local version of the dbCAN database (8, 9). A total of 280 predicted CAZymes were identified, including 113 glycoside hydrolases (GHs), 17 polysaccharide lyases (PLs), 58 carbohydrate esterases, 35 glycosyl transferases, 46 carbohydrate-binding modules, and 11 proteins with auxiliary activities. In addition, the genome contains 11 sulfatases and 51 TonB-dependent receptors, which have been implicated in carbohydrate scavenging (10). Besides agarases (CAZy families GH16, GH50, and GH86), the genome contains CAZymes with proposed function against other complex carbohydrates, such as carrageenans (GH16 and GH82), pectin (GH28, GH88, GH105, PL1, PL9, PL10, and PL11), alginate (PL6, PL7, PL14, and PL17), and xylan (GH10 and GH11). The large number of predicted CAZymes and associated functions suggests that *Paraglaciecola* sp. S66 can degrade and utilize a range of complex carbohydrates, including the sulfated polysaccharides found in algal cell walls.

### Nucleotide sequence accession numbers.

This whole-genome shotgun project has been deposited in DDBJ/ENA/GenBank under the accession number LSNE00000000. The version described in this paper is the first version, LSNE01000000.
